# After accounting for competing causes of death and more advanced stage, do Aboriginal and Torres Strait Islander peoples with cancer still have worse survival? A population-based cohort study in New South Wales

**DOI:** 10.1186/s12885-017-3374-6

**Published:** 2017-06-02

**Authors:** Hanna E. Tervonen, Richard Walton, Hui You, Deborah Baker, David Roder, David Currow, Sanchia Aranda

**Affiliations:** 1 School of Health Sciences, Centre for Population Health Research, University of South Australia, GPO Box 2471, Adelaide, SA 5001 Australia; 2 Information Analysis Unit, Cancer Institute NSW, GPO Box 41, Alexandria, Sydney, NSW 1435 Australia; 3 Cancer Institute NSW, GPO Box 41, Alexandria, Sydney, NSW 1435 Australia

**Keywords:** Neoplasms, Staging, Indigenous, Survival analysis, Australia, Epidemiology

## Abstract

**Background:**

Aboriginal and Torres Strait Islander peoples in Australia have been found to have poorer cancer survival than non-Aboriginal people. However, use of conventional relative survival analyses is limited due to a lack of life tables. This cohort study examined whether poorer survival persist after accounting for competing risks of death from other causes and disparities in cancer stage at diagnosis, for all cancers collectively and by cancer site.

**Methods:**

People diagnosed in 2000–2008 were extracted from the population-based New South Wales Cancer Registry. Aboriginal status was multiply imputed for people with missing information (12.9%). Logistic regression models were used to compute odds ratios (ORs) with 95% confidence intervals (CIs) for ‘advanced stage’ at diagnosis (separately for distant and distant/regional stage). Survival was examined using competing risk regression to compute subhazard ratios (SHRs) with 95%CIs.

**Results:**

Of the 301,356 cases, 2517 (0.84%) identified as Aboriginal (0.94% after imputation). After adjusting for age, sex, year of diagnosis, socio-economic status, remoteness, and cancer site Aboriginal peoples were more likely to be diagnosed with distant (OR 1.30, 95%CI 1.17–1.44) or distant/regional stage (OR 1.29, 95%CI 1.18–1.40) for all cancers collectively. This applied to cancers of the female breast, uterus, prostate, kidney, others (those not included in other categories) and cervix (when analyses were restricted to cases with known stages/known Aboriginal status). Aboriginal peoples had a higher hazard of death than non-Aboriginal people after accounting for competing risks from other causes of death, socio-demographic factors, stage and cancer site (SHR 1.40, 95%CI 1.31–1.50 for all cancers collectively). Consistent results applied to colorectal, lung, breast, prostate and other cancers.

**Conclusions:**

Aboriginal peoples with cancer have an elevated hazard of cancer death compared with non-Aboriginal people, after accounting for more advanced stage and competing causes of death. Further research is needed to determine reasons, including any contribution of co-morbidity, lifestyle factors and differentials in service access to help explain disparities.

## Background

Despite generally high standards of health care in Australia, health inequalities exist by socio-economic status, residential remoteness, migrant status and in particular, Aboriginal status [1]. Australian Aboriginal and Torres Strait Islander peoples (referred to in this article as Aboriginal peoples) experience mortality at a younger age and higher health morbidity compared with non-Aboriginal people [2, 3]. This disadvantage applies also to cancer, although the available evidence is limited by the incomplete recording of Aboriginal status on the data sources used by cancer registries, which may partially explain the reported lower cancer incidence among Aboriginal peoples [4]. Several studies have shown that Aboriginal peoples with cancer have lower survival compared with non-Aboriginal people [4–15] although the use of conventional relative survival analyses has been limited due to a lack of life tables. Cancer survival appeared to substantially improve for non-Aboriginal people in Australia in 1991–2005, but less so for Aboriginal peoples, which has widened the survival gap [8].

Probable reasons for differences in cancer survival include Aboriginal peoples being more likely to live in remote areas, having poorer access to screening and treatment services, receiving less optimal treatment and having higher levels of comorbidities [7, 10, 16]. In addition, available data indicate that Aboriginal peoples have a higher incidence of cancers with a poorer prognosis, reflecting differences in risk factor prevalence [17, 18]. Compared with non-Aboriginal people, Aboriginal peoples were more likely to be diagnosed with advanced stages for head and neck cancers [19], colon/rectum, breast, and cervix cancers, and non-Hodgkin lymphoma but not lung cancer [20]. Some studies have found lower survival among Aboriginal than non-Aboriginal people, even after adjustment for stage [4, 6, 9, 20], whereas other studies have indicated that the survival gap narrowed and became non-significant after adjustment for stage and other clinical factors [16] or after adjustment for comorbidities, socioeconomic disadvantage and remoteness [5]. The causes of survival disparities are complex, potentially geographically variable, and not fully understood. The possible effect of competing causes of death on survival estimates has not been investigated directly.

New South Wales (NSW) has the largest Aboriginal population in Australia, accounting for 30% of all Aboriginal peoples (overall 208,500 Aboriginal peoples lived in NSW in 2011) [21]. Previous studies from NSW have indicated that Aboriginal peoples have lower cancer survival than non-Aboriginal people (5-year survival 52.6% and 65.4% respectively for cases diagnosed in 1999–2007) [22]. A larger proportion of Aboriginal peoples were found to be diagnosed with distant stage than for non-Aboriginal people (19.3% vs. 13.5% for males; 19.2% vs. 14.5% for females). The NSW Cancer Registry (NSW CR) is the only Australian cancer registry routinely collecting stage (extent of disease) at diagnosis for all solid malignant tumours [23]. These data enable the simultaneous examination of differences in stage at diagnosis and survival.

After adjustment for stage, previous studies have reported lower survival for Aboriginal than non-Aboriginal people for cancers of the breast, prostate, lung, cervix, head and neck, stomach, pancreas and non-Hodgkin lymphoma [4, 5, 7, 9, 24] and conflicting results for colorectal cancer [9, 25]. Previous studies have generally examined either survival from all causes or disease-specific survival rather than using conventional relative survival due to the absence of credible life tables. Use of disease-specific mortality may be vulnerable to censoring bias and all cause survival masks the outcomes for cancer per se. To our knowledge, relative survival has only been used by Condon et al. (2014) for a period of 2001–2005 [8]. This study concluded that results from cause-specific and relative survival models were largely similar for all sites but there were differences in site-specific analyses. Our study takes a different approach by analysing mortality due to cancer taking competing causes into account. This is important because there is evidence that Aboriginal peoples with cancer are more likely to die from a non-cancer death than non-Aboriginal people [16].

The aim of this study was to examine whether poorer survival persists after accounting for competing risks of death from other causes and disparities in cancer stage at diagnosis, for all cancers collectively and by cancer site. We also report on the scale of disadvantage in cancer stage and survival experienced by Aboriginal peoples in the context of inequalities experienced by other population groups classified by socioeconomic status and remoteness of residence.

## Methods

### Study design and data sources

This cohort study used population-based data from the New South Wales Cancer Registry (NSW CR). The NSW CR receives legally mandated reports of all cases of primary invasive cancer (except non-melanoma skin cancers) diagnosed in NSW residents. The NSW CR is a case-based registry in which notifications relating to a particular cancer are linked to a single person. If the same person has another cancer, that cancer counts as a second case. This study included cases diagnosed between January 2000, the point at which Aboriginal status is regarded to have been more accurately recorded in NSW, and December 2008 [26].

The NSW CR data include demographic information, cancer diagnosis and death data, and residential address at diagnosis. Death data were obtained through the NSW Registry of Births, Deaths and Marriages and the Australian Bureau of Statistics (ABS). Death data included deaths due to cancer and deaths from other causes.

Approval for the study was obtained from the NSW Population and Health Services Research Ethics Committee (NSW PHSREC 2012 07410) and the Aboriginal Health and Medical Research (AH&MRC) ethics committee. To undertake this study, the respective data custodians for the NSW CR, the NSW Registry of Births, Deaths and Marriages, and the ABS provided approval to use each data set and to link records from the NSW Cancer Registry to each data set. Input was obtained from the NSW Cancer Institute’s Aboriginal Advisory Group for data and linkage projects.

### Measures

The main variable of interest was Aboriginal status which was derived from multiple information sources, including hospitals and the NSW Registry of Births, Deaths and Marriages. For the purposes of this study, and due to low numbers of Torres Strait Islander peoples, Aboriginal and Torres Strait Islander peoples were grouped together. Because of under-recording of Aboriginal status in health and death registries, we used multiple imputation (MI) to account for unknown Aboriginal status [4].

Cancer primary site was classified according to the International Classification of Diseases Oncology (ICD-O-3) [27]. The following classifications were used in this study: stomach (C16), colorectal (C18,C19-C21; separately also colon C18 and rectum C19-C21), liver (C22), pancreas (C25), lung (C33,C34), cutaneous melanoma (C44 with M872-M879), breast (C50), cervix (C53), uterus (C54,C55), prostate (C61), kidney (C64-C66,C68), bladder (C67), ill-defined & unspecified site & other rare cancers (C26,C39,C42,C48,C76,C80), and all other invasive cancer sites collectively that were not included in the specific categories. This grouping was used because it included the most common cancers among Aboriginal and non-Aboriginal people. Similar categorisation was used for classifying causes of cancer deaths by primary site. For non-cancer deaths, the NSW CR did not record the underlying causes of deaths.

Age was measured in years at time of cancer diagnosis. Age was categorised as 0–39, 40–49, 50–59, 60–64, 65–69, 70–74, 75–79, 80–84 and 85+ years, and expressed as a categorical variable in the analyses. Broader categorisation into <50, 50–69 and ≥70 years was used for age-stratified analyses. Sensitivity analyses with different age categorisations were conducted but results remained largely unchanged (data not shown).

Residential remoteness was based on the Accessibility/Remoteness Index of Australia (ARIA+) [28]. ARIA+ was based on measures of physical road distance between populated localities and the nearest service centres. Residential remoteness was categorised into major cities (reference category), inner regional, outer regional and remote/very remote areas.

Socio-economic status was estimated using the Index of Relative Socio-Economic Disadvantage (IRSD) based on residential data by ABS Statistical Local Areas at the time of diagnosis [29]. IRSD is one of the Socio-Economic Indexes for Areas (SEIFAs) created by the ABS. IRSD was categorised into quintiles (1: least disadvantaged (reference category) to 5: most disadvantaged).

Stage (extent of disease) is defined as the highest degree of spread based on all diagnostic and therapeutic evidence obtained within four months of cancer first being diagnosed according to international guidelines widely used by cancer registries worldwide [23, 30]. Stage was categorised as localised, regional, distant or unknown (if enough information to assign stage was not available).

### Statistical analyses

A MI model previously created by the NSW CR was modified for the purposes of this study [4, 22]. Logistic regression was used as a modelling approach to impute the values for cases with unknown Aboriginal status (*n* = 38,764, 12.9%). According to the missing at random (MAR) assumption, the probability of missingness can depend on the observed, but not on the missing data [31]. Therefore, MI model must include all predictors that are relevant to the missing-data mechanism [32]. Predictor variables included in the regression model were 5-year age group, sex, country of birth, stage at diagnosis, cancer site, one-year survival, Area Health Service of residence at diagnosis, SEIFA quintile, remoteness, year of diagnosis, and percentage of the local government area population identifying as Aboriginal. Use of several covariates as predictors of missing Aboriginal status is likely to make the MAR assumption tenable [4]. We imputed 20 datasets which were used in the analyses. MI estimates of coefficients and standard errors adjusted for the variability between imputations were computed using Rubin’s combination rules [33]. Cases with missing information with any of the predictor variables were excluded (*n* = 39). Sensitivity analyses excluding cases with missing Aboriginal status were also conducted.

Initially the study population was described using frequency distributions and cross-tabulations (both for complete-case and imputed data). Bi-variable associations between Aboriginal and non-Aboriginal people in complete-case data were explored using the Pearson chi-square test and Mann-Whitney (Wilcoxon rank-sum) test.

Logistic regression models were used to examine associations between Aboriginal status and stage of cancer at diagnosis for all cancers collectively and by cancer site, including cases with unknown stage. We also conducted sensitivity analyses excluding cases with unknown stage. Separate analyses were performed for distant and distant/regional stage, respectively, compared with other stage categories as the outcome variable and the term ‘advanced stage’ was used when referring to these outcomes. Multivariable models were fitted, adjusting for age, sex, year of diagnosis, remoteness and SEIFA quintile (model 1) and also cancer site (model 2). The effect of adding an interaction term for Aboriginal status and age to model 2 was examined using complete-case data. Results were presented as odds ratios (ORs) with 95% confidence intervals (CI).

Competing risk regression models using the Fine and Gray method were used to examine hazard of death due to cancer among Aboriginal compared with non-Aboriginal people for all cancers collectively and by site [34]. Competing risk regression models the subhazard function of an event of interest in the presence of competing events (also known as the cumulative incidence function). Deaths due to causes other than the cancer of diagnosis were regarded as competing events. Cases were followed from the time of diagnosis to death or to December 2008, which ever occurred first. Death certificate only (DCO) cases or cases found at post-mortem were excluded from survival analyses (*n* = 4406, 1.5%; a similar proportion affecting both Aboriginal and non-Aboriginal people, (Χ^2^_[df=1]_ = 2.7, *p* = 0.098). Multivariable models were adjusted for age, sex, year of diagnosis, remoteness and SEIFA quintile (model 1), stage (model 2) and cancer site (model 3). The effect of adding an interaction term for Aboriginal status and age to the final model was examined using complete-case data. Results were presented as subhazard ratios (SHRs) with 95%CIs. Final models were found to satisfy proportional hazards assumptions.

All analyses were performed using Stata Statistical Software: Release 12 (College Station, TX: StataCorp LP, 2011). Stata *stcrreg* command was used in survival analysis [35] and Stata *mi* commands were used in multiple imputation [32].

## Results

**Fig. 1:**
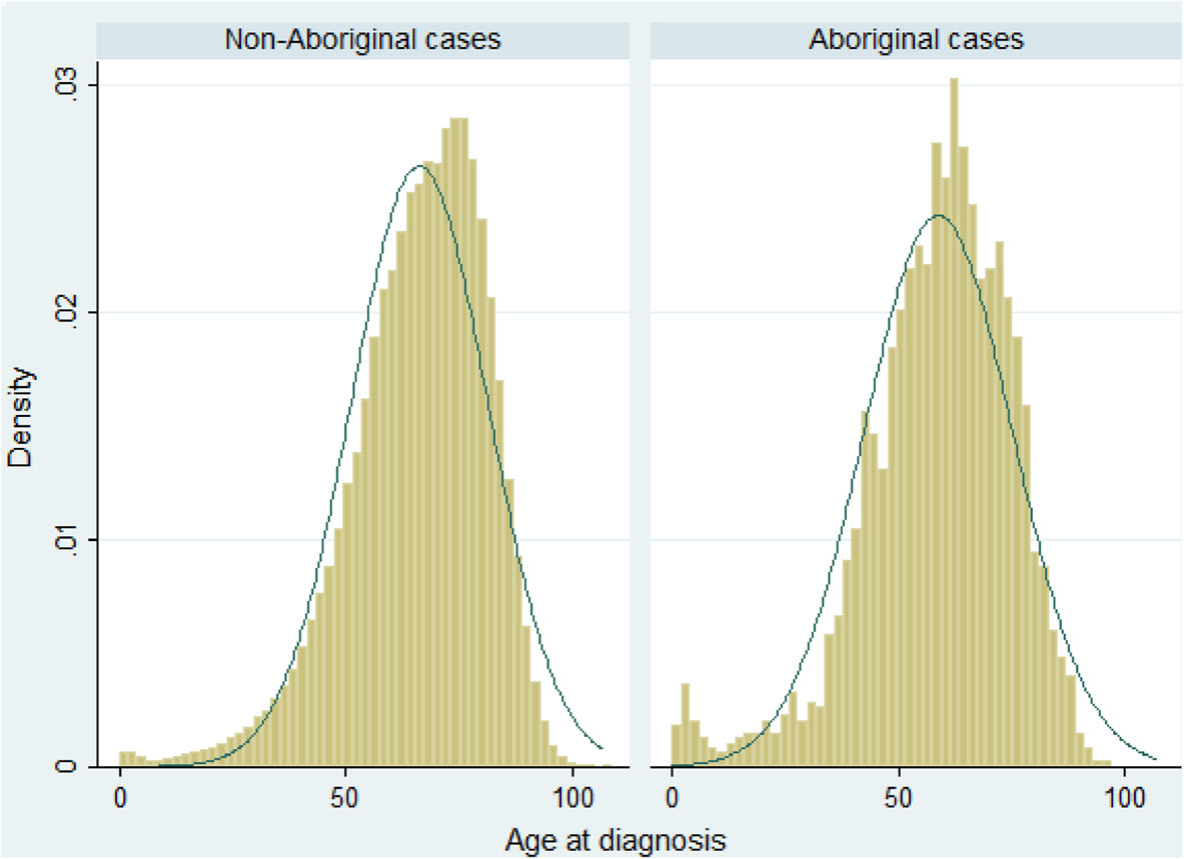
The age distributions at the time of diagnosis among non-Aboriginal and Aboriginal people

Altogether 301,356 cases with invasive cancer were diagnosed between 2000 and 2008 and followed for a mean duration of 2.8 years. Of these, 2517 (0.84%) were identified as Aboriginal and 38,764 (12.9%) had an unknown Aboriginal status. Aboriginal peoples were generally younger than non-Aboriginal people (median age 61 vs. 68 years) (Fig. 1). After imputation, the proportion of Aboriginal peoples increased from 0.84% to 0.94% (95%CI 0.90–0.98%) of all cases included into the analyses (compared to Aboriginal peoples accounting for 3.0% of Australia’s population). Characteristics of the study population are shown in Table 1.

**Table 1:** Characteristics of the study population overall and by Aboriginal status, NSW Cancer Registry 2000–2008

	All (*n* = 301,356)^a^	Aboriginal (*n* = 2517)		Non-Aboriginal (*n* = 260,075)		
	*n* (%)	*n* (CC %)	(MI %)^b^	*n* (CC %)	(MI %)^b^	*P* value^c^
Males	168,326 (55.9)	1313 (52.2)	(52.0)	144,109 (55.4)	(55.9)	Χ^2^_(1)_ *p* = 0.001
Age at diagnosis						Χ^2^_(8)_ *p* < 0.001; MW(z) = 24.3, *p* < 0.001
0–39	17,031 (5.7)	263 (10.5)	(11.0)	13,398 (5.2)	(5.6)	
40–49	24,538 (8.1)	360 (14.3)	(14.9)	19,810 (7.6)	(8.1)	
50–59	50,578 (16.8)	570 (22.7)	(22.7)	42,361 (16.3)	(16.7)	
60–64	34,754 (11.5)	358 (14.2)	(14.1)	29,730 (11.4)	(11.5)	
65–69	38,813 (12.9)	288 (11.4)	(11.3)	33,548 (12.9)	(12.9)	
70–74	40,688 (13.5)	290 (11.5)	(11.4)	35,587 (13.7)	(13.5)	
75–79	40,276 (13.4)	210 (8.3)	(7.9)	35,701 (13.7)	(13.4)	
80–84	30,592 (10.2)	103 (4.1)	(4.0)	27,658 (10.6)	(10.2)	
85+	24,086 (8.0)	75 (3.0)	(2.9)	22,282 (8.6)	(8.0)	
Residential remoteness						Χ^2^_(3)_ *p* < 0.001; MW(z) = −30.3, *p* < 0.001
Major cities	204,781 (68.0)	1099 (43.7)	(43.5)	178,436 (68.6)	(68.2)	
Inner regional	71,652 (23.8)	789 (31.4)	(31.2)	60,863 (23.4)	(23.7)	
Outer regional	23,235 (7.7)	493 (19.6)	(19.7)	19,467 (7.5)	(7.6)	
Remote/ Very remote	1688 (0.6)	136 (5.4)	(5.7)	1309 (0.5)	(0.5)	
SEIFA quintile						Χ^2^_(4)_ *p* < 0.001; MW(z) = −23.2, *p* < 0.001
1 (least disadvantaged)	62,971 (20.9)	192 (7.6)	(8.1)	54,552 (21.0)	(21.0)	
2	54,345 (18.0)	324 (12.9)	(13.0)	47,108 (18.1)	(18.1)	
3	61,341 (20.4)	484 (19.2)	(19.4)	52,517 (20.2)	(20.4)	
4	68,943 (22.9)	724 (28.8)	(28.3)	59,245 (22.8)	(22.8)	
5 (most disadvantaged)	53,756 (17.8)	793 (31.5)	(31.3)	46,653 (17.9)	(17.7)	
Stage at diagnosis						Χ^2^_(3)_ *p* < 0.001; MW(z) = −9.0, *p* < 0.001^d^
Localised	124,907 (41.5)	834 (33.1)	(34.7)	102,624 (39.5)	(41.5)	
Regional	55,210 (18.3)	578 (23.0)	(21.7)	51,572 (19.8)	(18.3)	
Distant	43,660 (14.5)	552 (21.9)	(19.8)	42,367 (16.3)	(14.4)	
Unknown	77,579 (25.7)	553 (22.0)	(23.8)	63,512 (24.4)	(25.8)	
Vital status^e^						Χ^2^_(2)_ *p* < 0.001
Alive	182,801 (60.7)	1172 (46.6)	(51.9)	144,476 (55.6)	(60.7)	
Died due to the cancer	89,465 (29.7)	1108 (44.0)	(39.6)	87,354 (33.6)	(29.6)	
Died due to other cause	29,090 (9.7)	237 (9.4)	(8.5)	28,245 (10.9)	(9.7)	
Cancer site						Χ^2^_(13)_ *p* < 0.001
Stomach	5794 (1.9)	73 (2.9)	(2.7)	5544 (2.1)	(1.9)	
Colorectal^f^	40,288 (13.4)	289 (11.5)	(11.0)	37,334 (14.4)	(13.4)	
Colon	25,794	176 (7.0)		23,832 (9.2)		
Rectum	14,494	113 (4.5)		13,502 (5.2)		
Liver	3433 (1.1)	50 (2.0)	(1.8)	3307 (1.3)	(1.1)	
Pancreas	6729 (2.2)	66 (2.6)	(2.3)	6539 (2.5)	(2.2)	
Lung	27,302 (9.1)	392 (15.6)	(14.2)	26,161 (10.1)	(9.0)	
Melanoma	30,166 (10.0)	70 (2.8)	(4.6)	16,966 (6.5)	(10.1)	
Breast^g^	37,266 (12.4)	331 (13.2)	(13.3)	32,754 (12.6)	(12.4)	
Cervix	2222 (0.7)	63 (2.5)	(2.6)	1908 (0.7)	(0.7)	
Uterus	5057 (1.7)	47 (1.9)	(1.8)	4620 (1.8)	(1.7)	
Prostate	48,071 (16.0)	226 (9.0)	(9.9)	38,712 (14.9)	(16.1)	
Kidney	8333 (2.8)	91 (3.6)	(3.6)	7478 (2.9)	(2.8)	
Bladder	6912 (2.3)	43 (1.7)	(1.6)	6527 (2.5)	(2.3)	
Ill-defined & unspecified & other rare	11,207 (3.7)	110 (4.4)	(4.1)	10,507 (4.0)	(3.7)	
Others^h^	68,576 (22.8)	666 (26.5)	(26.3)	61,718 (23.7)	(22.7)	

*CC* complete-case, *MI* multiple imputation, *MW* Mann-Whitney test, *SEIFA* socio-economic index for areas (Index of relative socio-economic disadvantage was used in this study).

^a^Includes also cases with unknown Aboriginal status.

^b^Percentage after imputing Aboriginal status.

^c^Χ^2^_(df)_ = Pearson Chi square test (degrees of freedom) was used to test categorical differences and MW(z) = Mann-Whitney test (z value) to test for ordinal differences between Aboriginal and non-Aboriginal people using complete-case data.

^d^Unknown category was excluded when Mann-Whitney test was conducted.

^e^Vital status at end of follow-up.

^f^Colorectal cancers grouped together and separately for colon (ICD-O-3 C18) and rectum cancers (ICD-O-3 C19-C21).

^g^ Includes 303 cases of male breast cancer.

^h^All other cancer sites categorised to this group.

### Stage at diagnosis

After adjustment for age, sex, remoteness, SEIFA and diagnostic year, Aboriginal peoples were more likely to be diagnosed with a distant stage compared with non-Aboriginal people (OR 1.59, 95%CI 1.45–1.75) (model 1) (Table 2). After further adjustment for site, the odds ratio decreased to 1.30 (95% CI 1.17–1.44) (model 2). Aboriginal status showed a stronger association with distant stage than did remoteness or SEIFA (OR for remote/very remote compared with major cities 1.02, 95%CI 0.89–1.16; OR for most compared with least disadvantaged SEIFA quintile 1.40, 95%CI 1.35–1.45). Results were similar when the outcome of interest was a diagnosis with distant/regional stage.

**Table 2:** Logistic regression analysis of factors associated with advanced stage at diagnosis, NSW Cancer Registry 2000–2008

	Distant			Distant/regional		
	Unadjusted	Model 1	Model 2	Unadjusted	Model 1	Model 2
	OR (95% CI)	AOR (95% CI)^a^	AOR (95% CI)^b^	OR (95% CI)	AOR (95% CI)^a^	AOR (95% CI)^b^
Aboriginal status						
Non-Aboriginal	1	1	1	1	1	1
Aboriginal	1.46 (1.33–1.61)	1.59 (1.45–1.75)	1.30 (1.17–1.44)	1.46 (1.34–1.58)	1.47 (1.36–1.60)	1.29 (1.18–1.40)
Sex						
Female	1	1	1	1	1	1
Male	0.91 (0.89–0.93)	0.88 (0.86–0.90)	0.91 (0.89–0.93)	0.66 (0.65–0.67)	0.65 (0.64–0.66)	0.96 (0.94–0.98)
Residential remoteness						
Major cities	1	1	1	1	1	1
Inner regional	0.94 (0.92–0.96)	0.87 (0.85–0.89)	0.91 (0.88–0.94)	0.92 (0.90–0.93)	0.89 (0.87–0.90)	0.92 (0.90–0.94)
Outer regional	1.00 (0.97–1.04)	0.90 (0.86–0.93)	0.94 (0.89–0.98)	0.96 (0.94–0.99)	0.91 (0.88–0.94)	0.95 (0.92–0.98)
Remote/ Very remote	1.19 (1.05–1.36)	1.02 (0.89–1.16)	0.97 (0.84–1.12)	1.09 (0.98–1.20)	0.98 (0.88–1.09)	0.97 (0.87–1.09)
SEIFA quintile						
1 (least disadvantaged)	1	1	1	1	1	1
2	1.17 (1.13–1.21)	1.21 (1.17–1.25)	1.10 (1.06–1.14)	1.07 (1.04–1.10)	1.10 (1.07–1.12)	1.02 (0.99–1.05)
3	1.17 (1.14–1.21)	1.22 (1.18–1.26)	1.07 (1.03–1.11)	1.05 (1.03–1.08)	1.10 (1.07–1.12)	1.00 (0.97–1.03)
4	1.17 (1.13–1.21)	1.23 (1.19–1.27)	1.07 (1.03–1.11)	1.05 (1.02–1.07)	1.11 (1.08–1.13)	1.01 (0.98–1.03)
5 (most disadvantaged)	1.32 (1.28–1.36)	1.40 (1.35–1.45)	1.17 (1.13–1.22)	1.15 (1.12–1.18)	1.21 (1.18–1.24)	1.05 (1.02–1.08)

*AOR* adjusted odds ratio, *CI* confidence interval, *OR* odds ratio, *SEIFA* socio-economic index for areas (Index of relative socio-economic disadvantage was used in this study).

^a^Model 1 adjusted for age, sex, year of diagnosis, remoteness and SEIFA.

^b^Model 2 further adjusted for cancer site.

In age-stratified analyses, the higher odds of being diagnosed with distant stage among Aboriginal compared with non-Aboriginal people tended to be more pronounced for people aged under 50 years (OR 1.51, 95%CI 1.20–1.91) compared with those aged 50–69 years (OR 1.18, 95%CI 1.02–1.38) or 70+ years (OR 1.24, 95%CI 1.02–1.51). However, the interaction between Aboriginal status and age was not statistically significant.

In cancer site-stratified analyses, an association between Aboriginal status and distant stage was observed for female breast (OR 1.62, 95%CI 1.11–2.36), uterus (OR 2.19, 95%CI 1.04–4.64), prostate (OR 2.59, 95%CI 1.65–4.08), kidney (OR 1.87, 95%CI 1.16–3.03) and other cancers (OR 1.63, 95% CI 1.30–2.04) (Table 3). When analysing distant/regional stage at diagnosis as the outcome, associations for uterus and prostate cancers attenuated. In addition, elevated odds of advanced stage among Aboriginal peoples were detected for colorectal cancer when distant/regional stage was the outcome (OR 1.34, 95%CI 1.05–1.70). Elevated odds were apparent for rectum cancer (OR 1.82, 95%CI 1.21–2.73) but not colon cancer.

**Table 3:** Site-stratified logistic regression models of the odds of advanced stage and competing risk regression models of the hazard of cancer death among Aboriginal compared with non-Aboriginal people, NSW Cancer Registry 2000–2008

Cancer site	Distant stage	Distant/regional stage	Hazard of cancer death
	AOR (95% CI)^a^	AOR (95% CI)^a^	SHR (95% CI)^b^
Stomach	1.01 (0.61–1.67)	0.93 (0.57–1.51)	1.23 (0.90–1.67)
Colorectal^c^	1.28 (0.97–1.69)	1.34 (1.05–1.70)	1.57 (1.32–1.87)
Colon	0.98 (0.68–1.42)	1.10 (0.81–1.50)	1.44 (1.13–1.84)
Rectum	1.93 (1.27–2.94)	1.82 (1.21–2.73)	1.78 (1.38–2.30)
Liver	0.66 (0.28–1.59)	0.69 (0.34–1.40)	1.27 (0.91–1.77)
Pancreas	0.83 (0.50–1.38)	0.78 (0.47–1.30)	1.16 (0.91–1.48)
Lung	0.94 (0.76–1.16)	0.95 (0.77–1.17)	1.39 (1.24–1.56)
Melanoma	1.51 (0.71–3.21)	1.61 (0.94–2.74)	1.11 (0.63–1.96)
Breast^d^	1.62 (1.11–2.36)	1.37 (1.10–1.71)	1.62 (1.22–2.16)
Cervix^d^	2.05 (0.96–4.37)	1.61 (0.94–2.74)	1.27 (0.80–2.03)
Uterus^d^	2.19 (1.04–4.64)	1.47 (0.80–2.70)	1.16 (0.67–2.02)
Prostate^d^	2.59 (1.65–4.08)	1.31 (0.90–1.90)	1.86 (1.24–2.77)
Kidney	1.87 (1.16–3.03)	1.55 (1.02–2.38)	0.89 (0.59–1.35)
Bladder	0.85 (0.26–2.79)	1.40 (0.74–2.66)	0.96 (0.57–1.62)
Ill-defined & unspecified & other rare	0.99 (0.64–1.53)	0.83 (0.51–1.34)	1.26 (0.99–1.61)
Others^e^	1.63 (1.30–2.04)	1.52 (1.28–1.80)	1.40 (1.23–1.59)

*AOR* adjusted odds ratio, *CI* confidence interval, *SHR* sub-hazard ratio, *ICD-O-3* International Classification of Diseases Oncology, *SEIFA* socio-economic index for areas (Index of relative socio-economic disadvantage).

^a^All logistic regression models adjusted for age, sex, year of diagnosis, remoteness and SEIFA. ORs presented for Aboriginal peoples compared with non-Aboriginal people. Separate models for distant and distant/regional as an outcome.

^b^All competing risk regression models adjusted for age, sex, year of diagnosis, remoteness, SEIFA and stage. SHRs presented for Aboriginal peoples compared with non-Aboriginal people. Death certificate only cases and cases found at post-mortem were excluded from survival analysis (1.5%) and, therefore, numbers included in stage and survival analyses differ slightly.

^c^Colorectal cancers grouped together and separately for colon (ICD-O-3 C18) and rectum cancers (ICD-O-3 C19-C21).

^d^Only females/ males included as relevant. Only female breast cancers included in the models.

^e^All other cancer sites categorised to this group.

Sensitivity analyses excluding cases with unknown stage produced largely similar risk estimates for Aboriginal status (data not shown), with the exception of a stronger association between Aboriginal status and advanced stage for cervix cancer (distant stage OR 2.24, 95%CI 1.04–4.80; distant/regional stage OR 1.79, 95%CI 1.01–3.17 for Aboriginal compared with non-Aboriginal people). Results were similar when those with missing Aboriginal status were excluded from the analyses.

### Survival

Competing risk regression modelling, adjusted for age, sex, year of diagnosis, remoteness and SEIFA, indicated that Aboriginal peoples had an elevated hazard of death from the cancer compared with non-Aboriginal people (SHR 1.76, 95%CI 1.65–1.88) (Table 4). After further adjustment for stage, the subhazard ratio decreased to 1.54 (95%CI 1.44–1.65) and then further to 1.40 (95%CI 1.31–1.50) after adjusting for cancer site.

**Table 4:** Competing risks regression models of factors associated with survival, NSW Cancer Registry 2000–2008

	Unadjusted model	Model 1^a^	Model 2^b^	Model 3^c^
	SHR (95% CI)	SHR (95% CI)	SHR (95% CI)	SHR (95% CI)
Aboriginal	1.47 (1.38–1.57)	1.76 (1.65–1.88)	1.54 (1.44–1.65)	1.40 (1.31–1.50)
Male	1.07 (1.05–1.08)	1.01 (0.99–1.02)	1.07 (1.05–1.08)	1.07 (1.05–1.08)
Age at diagnosis				
0–39	1	1	1	1
40–49	1.52 (1.44–1.60)	1.52 (1.45–1.61)	1.39 (1.32–1.46)	1.54 (1.47–1.63)
50–59	1.97 (1.88–2.07)	2.00 (1.91–2.10)	1.78 (1.70–1.86)	1.93 (1.84–2.02)
60–64	2.26 (2.15–2.37)	2.30 (2.19–2.41)	2.01 (1.92–2.11)	2.14 (2.04–2.25)
65–69	2.54 (2.43–2.67)	2.58 (2.46–2.70)	2.25 (2.15–2.36)	2.36 (2.25–2.48)
70–74	3.23 (3.09–3.38)	3.25 (3.10–3.40)	2.73 (2.61–2.86)	2.80 (2.67–2.94)
75–79	3.79 (3.62–3.97)	3.84 (3.67–4.02)	3.19 (3.04–3.34)	3.26 (3.10–3.42)
80–84	4.59 (4.39–4.81)	4.72 (4.50–4.94)	3.89 (3.71–4.08)	4.01 (3.82–4.21)
85+	5.78 (5.52–6.06)	5.99 (5.71–6.28)	4.84 (4.61–5.08)	5.20 (4.95–5.47)
Residential remoteness				
Major cities	1	1	1	1
Inner regional	1.01 (1.00–1.03)	0.92 (0.91–0.94)	0.96 (0.94–0.98)	1.02 (1.00–1.04)
Outer regional	1.06 (1.03–1.09)	0.94 (0.92–0.97)	0.98 (0.96–1.01)	1.05 (1.02–1.08)
Remote/ Very remote	1.12 (1.03–1.22)	0.99 (0.91–1.08)	0.96 (0.87–1.05)	1.01 (0.92–1.11)
SEIFA quintile				
1 (least disadvantaged)	1	1	1	1
2	1.16 (1.13–1.18)	1.19 (1.17–1.22)	1.12 (1.10–1.15)	1.09 (1.06–1.11)
3	1.22 (1.20–1.25)	1.25 (1.22–1.28)	1.18 (1.15–1.21)	1.14 (1.12–1.17)
4	1.27 (1.25–1.30)	1.29 (1.27–1.32)	1.22 (1.19–1.25)	1.17 (1.15–1.20)
5 (most disadvantaged)	1.30 (1.27–1.32)	1.38 (1.35–1.41)	1.24 (1.21–1.27)	1.15 (1.12–1.18)
Stage				
Localised	1	−	1	1
Regional	2.43 (2.38–2.48)	−	2.40 (2.35–2.46)	1.92 (1.88–1.96)
Distant	11.08 (10.87–11.29)	−	10.32 (10.12–10.53)	5.59 (5.47–5.72)
Unknown	2.45 (2.40–2.50)	−	2.15 (2.11–2.19)	1.60 (1.56–1.64)

*SHR* subhazard ratio, *CI* confidence interval, *SEIFA* socio-economic index for areas (Index of relative socio-economic disadvantage).

^a^Model 1 adjusted for age, sex, year of diagnosis, remoteness and SEIFA

^b^Model 2 further adjusted for stage at diagnosis.

^c^Model 3 further adjusted for cancer site.

An interaction term for Aboriginal status and age indicated varying effects in different age groups. In age-stratified analyses, Aboriginal peoples aged less than 50 years tended to have a higher elevated relative risk of death from the cancer compared with non-Aboriginal people (SHR 1.65, 95%CI 1.41–1.93) than those aged 50–69 (SHR 1.45, 95%CI 1.32–1.60) and 70+ years (SHR 1.15, 95%CI 1.02–1.29).

After adjustment for demographic factors and stage, site-stratified analyses showed an elevated hazard of death from the cancer for Aboriginal peoples for colorectal (SHR 1.57, 95%CI 1.32–1.87), lung (SHR 1.39, 95%CI 1.24–1.56), breast (SHR 1.62, 95%CI 1.22–2.16), prostate (SHR 1.86, 95%CI 1.24–2.77) and other cancers (SHR 1.40, 95%CI 1.23–1.59) (Table 3).

Results remained similar when cases with unknown Aboriginal status were excluded.

## Discussion

This is one of the largest studies examining cancer stage and stage-adjusted survival disparities among Aboriginal and non-Aboriginal people in Australia, made possible by routine recording of stage by the NSW CR. The main finding of this study was that after accounting for competing causes of death and more advanced stage, Aboriginal peoples with cancer still had worse survival than non-Aboriginal people. Our results also indicate that Aboriginal status is a stronger predictor of advanced stage and hazard of cancer death than living in remote or socio-economically disadvantaged areas, as classified in this study. Indigenous populations worldwide face similar disparities which are shaped by historical process of colonisation, marginalisation, dislocation, trauma and the absence of recognition [36, 37]. Therefore, social determinants, referring to historical, political, economic and social contexts into which people are born, may be especially important for health outcomes, including cancer outcomes, of Aboriginal peoples [38, 39].

Aboriginal peoples were more likely to be diagnosed with an advanced stage compared with non-Aboriginal people. Previous studies have similarly reported that Aboriginal peoples had more advanced stage [4–6, 20] but to our knowledge only one previous study in addition to ours has systematically examined cancer site-specific differences [19]. Age-stratified analyses indicated that the association between Aboriginal status and advanced stage tended to be stronger in younger age groups, however, interaction was not statistically significant. The reasons for this finding are not known, although it may be explained by older peoples having more contact with the health care system, and thus experiencing more opportunities for clinical detection of cancer, irrespective of Aboriginal status. Another possibility is that older Aboriginal peoples may be more health-conscious and more inclined to respond to symptoms because they are a select group of people who have already survived to that age. Reasons behind the association between Aboriginal status and advanced stage of cancer are likely to reflect a complex interplay of both individual (awareness of symptoms, reluctance to seek treatment due to a lack of culturally appropriate services, participating in screening) and system level factors (access to health care services) [9]. Qualitative research is needed to explore these reasons.

In terms of both distant and distant/regional stage, the association between Aboriginal status and advanced stage was detected for breast, kidney and other cancers. The association for breast cancer may be partly explained by Aboriginal peoples participating in screening and other early detection initiatives less frequently than non-Aboriginal people [2, 10]. A similar association was less clear for other cancers addressed by screening, such as cervical cancer, although increased odds of advanced-stage cervical cancer in Aboriginal women were found when cases with an unknown stage/ unknown Aboriginal status were excluded. Population-based screening programs in Australia have not been able meet the needs of priority population groups, such as Aboriginal peoples, but future opportunities for improvement exist [40]. In terms of kidney cancer, imaging tests needed for detecting small tumours are expensive and centralised in major specialist centres, and therefore, possibly less accessible to Aboriginal peoples.

Relative survival is the most commonly used method to measure survival in population-based cancer studies but a lack of detailed life tables limits the use of this methodology for many population sub-groups [8, 41, 42]. Also life tables may not be relevant to smaller sub-groups within these populations, such as cases with advanced stage or those with a defined mix of socio-demographic characteristics. Net survival (cause-specific survival and relative survival) is the probability of surviving in the hypothetical world where the cancer under study is the only possible cause of death (i.e., in the absence of other causes of death). Net survival does not provide a measure of the true probability that a patient will die of their cancer. As there is evidence that Aboriginal peoples with cancer are more likely to die from a non-cancer death than non-Aboriginal people [16], it is useful to estimate the probability of cancer death in the presence of other causes. Therefore, we chose in this study to examine cumulative incidence of cancer deaths by conducting competing risk regression analyses. Competing risk regression modelling calculates the cumulative incidence of the cancer death under study in the presence of other causes of deaths. To our knowledge, no previous studies of survival among Aboriginal peoples have used this method. An advantage is that relevant population life tables are not needed. A disadvantage is reliance on attribution of cause of death in the NSW CR, the accuracy of which may be uncertain. We defined the cancer cause of death as a death matching specifically the diagnosed cancer. Future studies should examine the impact of using a broader definition of cancer death, e.g., as suggested by the National Cancer Institute [43]. A broader definition of cancer death would have decreased the proportion of competing causes of deaths and consequently generally decreased risk estimates. Therefore, our approach may have underestimated the hazard of cancer death.

Our results indicate that Aboriginal peoples have poorer survival from cancer than non-Aboriginal people, which is consistent with results of studies using cause-specific survival [4, 5, 16]. Such differences are multi-factorial and reflect differences across the spectrum of cancer control. Aboriginal peoples were more likely to be diagnosed with poor prognosis cancers (e.g., lung cancer) and less likely to be diagnosed with good prognosis cancers (e.g., melanoma and prostate cancer). Nevertheless, survival disparities remained even after adjusting for cancer site. An elevated hazard of death from the cancer tended to be more pronounced in Aboriginal peoples in the younger age groups. Similarly, a previous study reported higher elevations in cancer mortality for Aboriginal compared with non-Aboriginal people in younger than older people [44].

An elevated hazard of death after adjustment for demographic factors and stage was detected for colorectal, lung, breast, prostate and other cancers. Previous studies utilizing cause-specific survival models have reported similar results for a number of cancer sites [4, 5, 7, 9, 10, 24] but also differing results for colorectal cancer [25]. Cancer survival disparity seems to be only partly explained by differences in stage. Treatment-related factors, such as access to and quality of culturally appropriate treatment, and comorbidities are likely to play important roles [5, 16, 24]. Poorer outcomes for Aboriginal peoples may be due to different factors for different cancers. For example, worse lung cancer survival among Aboriginal peoples may be explained mostly by treatment differences and to a lesser extent by comorbidities [7]. Any differences in rates of treatment uptake and completion need to be quantified carefully. Similar proportions of DCO/post-mortem cases among Aboriginal and non-Aboriginal people indicate that both Aboriginal and non-Aboriginal people are responding to symptoms.

### Limitations and strengths

We did not have information on individual-level factors, such as life-style related risk factors, co-morbidities or participation in screening, which are likely to differ between Aboriginal and non-Aboriginal peoples and have impact on stage and survival. Socio-economic status and remoteness were based on area-level measurements at the time of diagnosis and may have changed during the follow-up period. Our study included people diagnosed in 2000–2008 and, therefore, cannot provide information about more recent trends. The mean follow-up time was relatively short. Strengths of the present study included population-based data and the use of MI to address the under-recording of Aboriginal status. After imputation, 0.94% of cases were identified as Aboriginal which is close to the national estimate (1% of new cancer cases being Aboriginal) [45]. Nevertheless, this is still likely to be an underestimate due to under-recording of Aboriginal status, as Aboriginal peoples account for 3% of the Australian population [21]. Previous studies which have used complete-case data may have underestimated the proportion of Aboriginal peoples. In addition, deaths due to competing events were taken into account which is important because in general Aboriginal peoples face higher mortality burden than non-Aboriginal people [2].

## Conclusions

After accounting for competing causes of death and more advanced stage, Aboriginal peoples had an elevated hazard of death from cancer compared with non-Aboriginal people. Active steps are needed to better understand reasons for these inequalities, especially in relation to preventable cancers, through qualitative research. We consider that effects on outcomes of co-morbidity and poorer service access and treatment should be a main focus of future quantitative research.

## Abbreviations

ABS: Australian Bureau of Statistics
ARIA: Accessibility/Remoteness Index of Australia
CI: Confidence interval
DCO: Death certificate only
ICD-O-3: International Classification of Diseases Oncology (3rd edition)
IRSD: Index of Relative Socio-Economic Disadvantage
MAR: Missing at random
MI: Multiple imputation
NSW CR: New South Wales Cancer Registry
NSW: New South Wales
OR: Odds ratio
SEIFA: Socio-Economic Index for Areas
SHR: Subhazard ratio
